# Corrosion Resistance of L120G13 Steel Castings Zone-Reinforced with Al_2_O_3_

**DOI:** 10.3390/ma15124090

**Published:** 2022-06-09

**Authors:** Daniel Medyński, Jacek Chęcmanowski

**Affiliations:** 1 Faculty of Technical and Economic Sciences, Witelon Collegium State University, Sejmowa 5A, 59-220 Legnica, Poland; 2 Department of Advanced Material Technologies, Wroclaw University of Science and Technology, Wybrzeże Wyspiańskiego 27, 50-370 Wroclaw, Poland; jacek.checmanowski@pwr.edu.pl

**Keywords:** cast steel, ceramics, composite, corrosion research, corrosion resistance, SEM research

## Abstract

The aim of the study was to determine the effect of zone reinforcement of cast steel L120G13 with Al_2_O_3_ macro-particles on the corrosion resistance of the composite obtained in this way. The obtained results allow us to conclude that strengthening of cast steel with corundum, the aim of which was to significantly increase the abrasive wear resistance, did not significantly deteriorate corrosion resistance. SEM tests show that a permanent diffusion layer interface is formed at the boundary between cast steel and corundum. In this area, simple manganese segregation and reverse iron and chromium segregation were found. These elements pass from the liquid alloy to the surface layer of the corundum particles, causing the aluminium and oxygen to be pushed deep into the corundum grains. Corrosion tests indicate comparable corrosion resistance of cast steel L120G13 and the composite L120G13 + Al_2_O_3_. Moreover, no intergranular corrosion was found in the matrix of the composite and no signs of pitting corrosion were found in the areas of the interface between the phases of the composite. This information is extremely important from the point of view of the material’s service life. Observations of breakthrough of both materials obtained during fracture after potentiodynamic corrosion tests, immediately after freezing in liquid nitrogen, indicate cracking with plastic features and increased resistance to dynamic forces of cast steel L120G13 and the composite L120G13 + Al_2_O_3_.

## 1. Introduction

Composite materials are hybrid materials, consisting of at least two materials with different properties. One of them is the matrix, the other is the reinforcement [1,2,3,4,5,6,7]. The matrix can be a metallic or non-metallic material. The reinforcement may also be a metallic or non-metallic material, and the reinforcement may take various forms, e.g., fibres, filings, sheets or solid particles such as e.g., oxides (Al_2_O_3_) or carbides (SiC). These phases are introduced to increase the mechanical properties and/or operational properties (e.g., abrasion resistance) of the base material [8,9,10]. Due to this, composites are most often classified according to the type of reinforcing phases. The first type is composites with continuous reinforcement elements such as long sheets, glass or carbon fibres. The second type are composites with discontinuous reinforcing elements such as particles, whiskers (thread crystals), filings, short fibres, etc. Moreover, composites of this type can be more easily shaped during possible secondary processing, such as forging or rolling [11]. Nowadays, a wide spectrum of composites are used, many of which are materials obtained in casting processes [11]. Literature reports indicate that casting reinforcement is most often used in the case of non-ferrous metal alloys, e.g., aluminium alloys [12]. It consists of making porous inserts of particles or Al_2_O_3_ or SiC fibres, and then their pressure infiltration with a liquid alloy [13,14,15]. For this reason, this method cannot be applied to the production of large steel or iron castings. In this case, the methods of surface impregnation of castings with alloying elements or the creation of surface composite layers with the technology of bimetallic layered castings are used [16,17,18,19]. However, the layer thicknesses of increased resistance to abrasion obtained by these methods do not exceed a few millimetres. In the mining industry, the abrasive wear of working machine elements is at least an order greater, and this should also be the thickness of the casting strengthening zone. An example of such a deep strengthening of steel castings is presented in [20]. As a result of the use of corundum fittings, strengthening was achieved in those places of the casting that are exposed to intense abrasion, leaving in the remaining areas the high mechanical properties of the base cast steel. Thus, a composite was obtained that combined the advantages of Hadfield cast steel (high strength, impact tough as well abrasive wear resistance) and corundum (very high hardness and abrasive wear resistance) [21].

Working elements of mining machines are exposed not only to intense abrasive wear and strong dynamic loads, they are also exposed to a strong influence of an aggressive corrosive environment, especially during downtimes. In order to increase the corrosion resistance of Fe-C alloys, corrosion protection layers are created on their surface, using various methods of surface impregnation [22,23,24]. Their thickness usually does not exceed a few millimetres. For this reason, they cannot be used for working elements of mining machines, as their wear reaches even a few centimetres. Elements that guarantee a single-phase structure are often introduced into the alloys, as it is known that such alloys exhibit the highest corrosion resistance. For this reason, composites (increased working properties), joining diametrically opposed materials, are usually less resistant to corrosion. If so, how much? The authors of this publication have attempted to answer this question.

## 2. Materials and Methods

**Figure 1:**
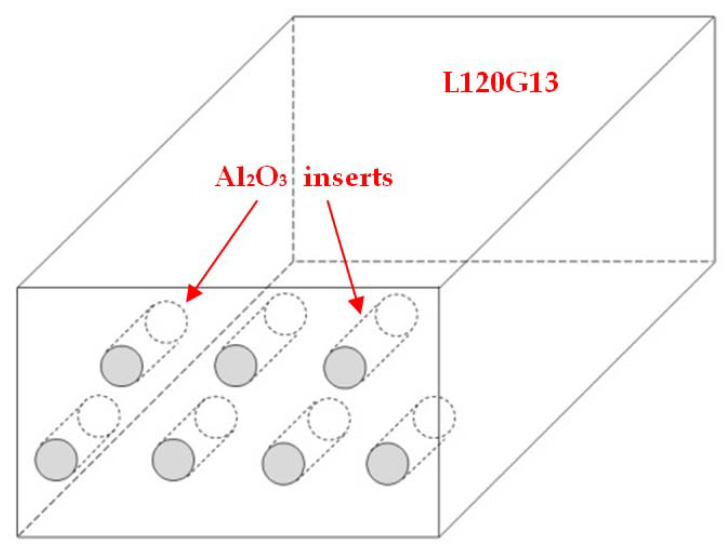
Diagram showing the method of reinforcement of castings.

The subject of the tests are castings made of Hadfield cast steel L120G13 (according to PN-89/H-83160, GX120Mn13 ISO, A128 ASTM standards), zone-reinforced, several centimetres from the reinforcement front, with ordinary corundum (Al_2_O_3_) particles, according to the diagram shown in Figure 1.

Increased strength and abrasive wear resistance are positively influenced by permanent connections between the composite components. Permanent connections prevent the corundum particles from cracking under the influence of external forces and their crushing during friction. However, the connections between the composite components may be weakened by corrosion, therefore various corrosion tests have been carried out. Additionally, a series of microscopic observations and analyses of the chemical composition at the interface between the two phases of the composite were carried out before and after the corrosion tests.

Microscopic observations were made using an OZL 963 light stereoscopic microscope (Kern Optics, Stuttgart, Germany), a TM 3000 scanning electron microscope (Hitachi, Tokyo, Japan) and a Quanta 250 (FEI, Hillsboro, OR, USA).

The analysis of the chemical composition of the matrix of the tested samples was performed using the spectral method using the GDS 750 QDP fluorescent analyser (Leco, St. Joseph, MI, USA) and X-ray energy dispersion spectroscopy method (SEM Quanta 250 FEI with EDS detector). The WDS detector was also used for comparative purposes.

Corrosion tests were carried out with the gravimetric and potentiometric methods. A 3% aqueous NaCl solution was used as the corrosive environment [25,26,27,28]. During gravimetric measurements, in order to increase the aggressiveness of the 3% NaCl solution, its aeration was used. The gravimetric tests consisted of determining the corrosion rate on the basis of the material mass loss in relation to the surface unit as a function of time. The linear corrosion rate V_P_ was determined from the relationship [29,30]:

V_P_ = 0.0365 × V_C_/d (mm/year), (1)

where:

V_C_—loss of sample mass over time (mg/m^2^·day),

d—metal material density [g/cm^3^], for cast steel L120G13 it was assumed 7.9 (g/cm^3^) [21].

Gravimetric tests were carried out in the Corr-Eco 108 salt chamber (Corr-Lab, Zgierz, Poland), while the potentiodynamic tests were performed in a three-electrode system, which consisted of a measuring cell and an SP-300 potentiostat (BioLogic, Seyssinet-Pariset, France).

The potential was determined in relation to the saturated calomel electrode, the auxiliary electrode was a platinum electrode. In each case, the potentiodynamic tests were conducted with the anodic polarisation at the rate of 1 mV/s. Corrosion resistance was determined on the basis of the following electrochemical parameters: cathode-anode transition potentials (E_K-A_), open cell potential (E_OC_), as well as corrosion current density (i_corr_) and polarisation resistance (R_p_) [26,27,28,29,30].

The examination of surface topography of samples was carried out using the CV-3200 device (MITUTOYO, Kawasaki, Japan), which was calibrated as follows: measuring length (X) 15.0000 mm, measuring step 0.0005 mm, measuring speed 1.00 mm/s, axis range (Z) 0.800 mm. Measurements were made in accordance with EN ISO 4287: 1998/AC: 2008 and PN-EN ISO 4288: 2011. During the tests, the following parameters were determined: average height of the highest hill of the profile (R_pAVR_), average depth of the lowest profile depression (R_vAVR_), mean absolute value of the five highest elevations and the five lowest depressions (R_zAVR_).

All tests were carried out on at least three properly prepared samples from different castings.

## 3. Results and Discussions

### 3.1. Microscopic Observations of Samples from Raw Castings

**Figure 2:**
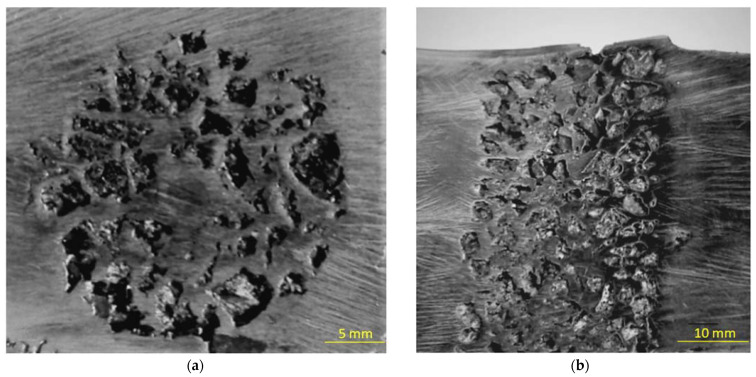
The fragment of the casting surface: (**a**) perpendicular, (**b**) parallel to axis of reinforcement (light microscopy).

The macroscopic observations showed that the ceramic particles were fully infiltrated with liquid metal during casting in the reinforcement zones. This is documented by the photos of the fragments of surfaces perpendicular and parallel to the reinforcement axis of the casting presented in Figure 2. In both cases, the complete continuity of the structure is visible at the interface between the matrix and the ceramic particles.

**Figure 3:**
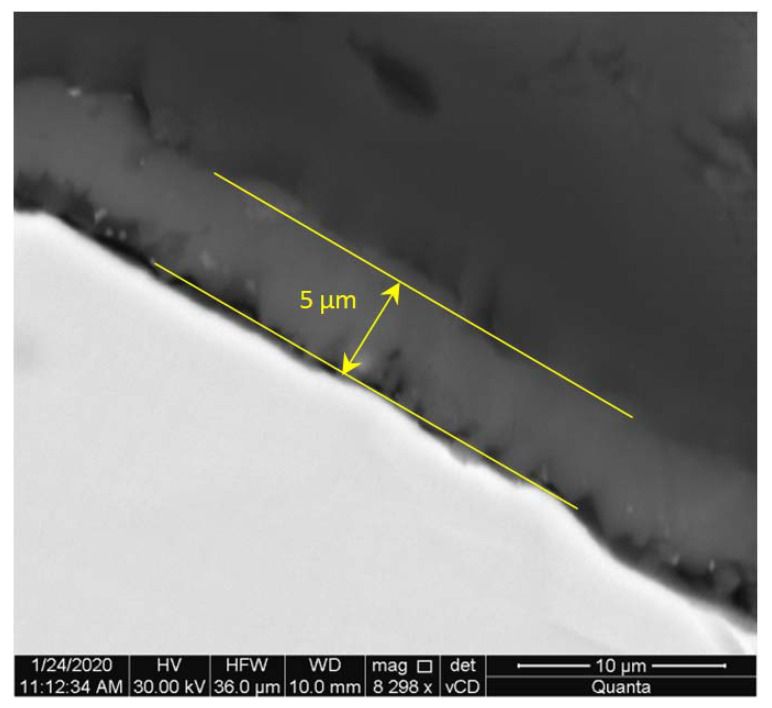
The layer at surface of corundum grain in the composite (before exposure to a 3% aqueous NaCl solution).

Figure 3 presents SEM photos showing the contact area of both phases. A thin layer approximately 5 µm thick was observed at the surface of the corundum particles. The layer is formed as a result of diffusion processes taking place during casting.

**Figure 4:**
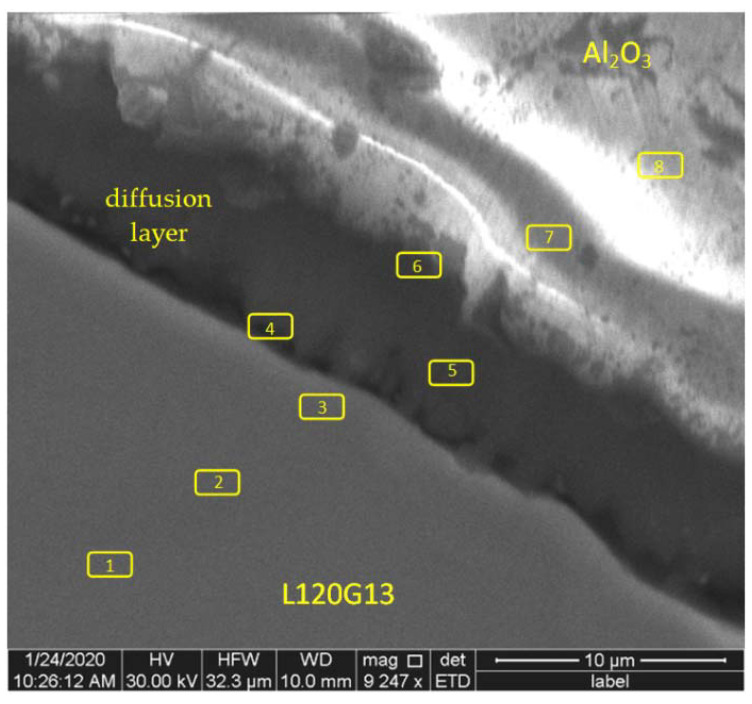
The area of boundary between phases of the composite with marked EDS measurement places.

During further research an attempt has been made to explain the formation of this layer. A series of surface measurements of the chemical composition were carried out in the boundary areas of both phases. Figure 4 presents a photo showing the contact area of the composite components with the marked places of chemical composition measurements using the EDS method. The WDS analysis was also carried out for comparative purposes. The results of EDS and WDS were similar, therefore Table 1 contains only the results of the EDS measurements.

It was found that some elements are segregated—Table 1. In the area of the diffusion zone manganese shows simple segregation, while iron and chromium show reverse segregation (lines marked in gray).

These elements pass from the liquid alloy to the surface layer of corundum particles, pushing aluminium and oxygen inside the corundum grains. This is confirmed by the values of the ratio of aluminium concentration to oxygen concentration Al/O—Table 1.

**Figure 5:**
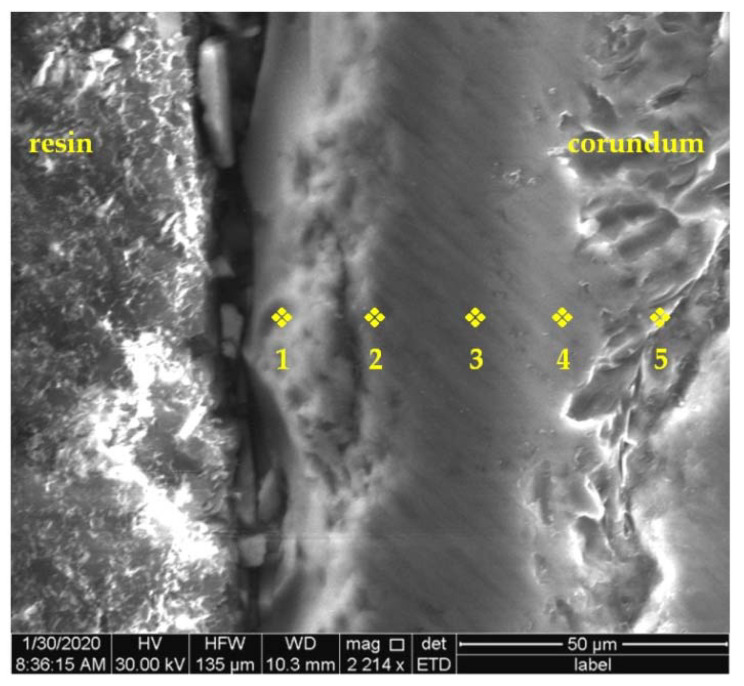
The corundum grain embedded with resin with EDS measurement points marked.

For comparative purposes, a reference sample was prepared in the form of corundum grains that had been previously embedded with resin. The sample was polished, and then microscopic observations and measurements of the chemical composition distribution on the cross-sectional surface of the corundum particles were performed. There was no subsurface layer around the corundum grains, contrary to the layer observed in the composite (Figure 5). The decomposition of aluminium and oxygen was similar both inside and at the grain surface. The results of the chemical composition analysis are presented in Table 2.

In addition, the corundum grains were subjected to a high temperature (1450 °C) in the same way as during the smelting process. After cooling down, the grains were included. After polishing, the samples were subjected to microscopic observations and EDS analysis. Additionally in this case, no subsurface layer was found around the corundum grains. The decomposition of aluminium and oxygen was similar both inside and at the grain surface.

### 3.2. Corrosion Tests

In order to determine the corrosion resistance of cast steel L120G13 and the composite L120G13 reinforced with Al_2_O_3_, these materials were subjected to corrosion tests using two test methods: gravimetric and potentiodynamic.

#### 3.2.1. Gravimetric Measurements

Gravimetric measurements were carried out for a period of 25 days. The samples were weighed (after prior cleaning) after the following holding times in a 3% aqueous NaCl solution: 1, 2, 5, 8, 13, 18 and 25 days. The corrosion rate of the castings as a function of time was determined using the Equation (1). The test results are summarised in Table 3.

Gravimetric tests revealed slight differences in corrosion resistance between the steel casting and the steel casting reinforced with corundum during exposure to a corrosive solution (Table 1). After 1 day of storing the samples in a corrosive environment, the corrosion rate of raw castings was in the range of 0.56 ÷ 0.59 mm/year. Extending the exposure time of the samples to 2 days did not cause any significant changes in the corrosion rate of both materials. The longer storage of the samples (more than 2 days) in the corrosive solution resulted in a gradual reduction of the corrosion rate. The formed corrosion products limited the access of the corrosive solution to the surface of the samples, causing their “screening”. This phenomenon is very beneficial from the point of view of corrosion resistance and durability of metallic materials. After 25 days of exposure of the samples in a corrosive solution, the corrosion rate in both cases was 0.36–0.38 mm/year. It was found that the corrosion rate decreased by about 30–40% in relation to the initial values. The gravimetric tests show that the introduction of corundum grains into the L120G13 cast steel does not significantly deteriorate the corrosion resistance determined by the gravimetric method.

#### 3.2.2. Potentiodynamic Measurements

During potentiodynamic tests, the samples were polarised after various times of their holding in a 3% aqueous NaCl solution (Table 4). In order to assess the corrosion resistance of the materials, the time of their exposure to the corrosive agent was gradually extended, up to 144 days. The measurement results are presented in Table 4.

Potentiodynamic tests of the cast steel L120G13 and the composite L120G13 containing Al_2_O_3_ particles after a 15-min exposure to 3% NaCl are a reference point for the preservation of these materials during the prolonged operation of a corrosive solution (Table 4). Small differences (approx. 20 mV) in the values of the open cell potential (E_OC_) between cast steel and cast steel composite indicate that the introduction of Al_2_O_3_ into the matrix does not change the “nobility” of the L120G13 surface. Additionally, with such a small difference in Eoc potential between cast steel and composite, there is a small chance of the formation of a corrosion link at the interface L120G13—Al_2_O_3_ (Table 4).

After a 1-day exposure of the samples in 3% NaCl, on the basis of the obtained results, it was found that the corrosion processes occurring on the surface of cast steel and composite are of a similar nature. In both cases, these processes were similar, but there were differences in the changes in individual electrochemical parameters (Table 4). Significant changes in the value of the open cell potential (E_OC_) by approx. 100 mV occur mainly for cast steel, especially in the initial phase of exposure of the samples in solution, i.e., after 1 day (Table 4). During the prolonged exposure of L120G13 samples to 3% NaCl (up to 144 days), the changes in E_OC_ values were small. In turn, for the composite, the changes in E_OC_ values were small (approx. 40 mV) during the entire exposure of this material in a corrosive solution (Table 4). Such small differences in the values of this potential indicate the “stability” of the surface exposed to the aggressive action of chlorides.

**Figure 6:**
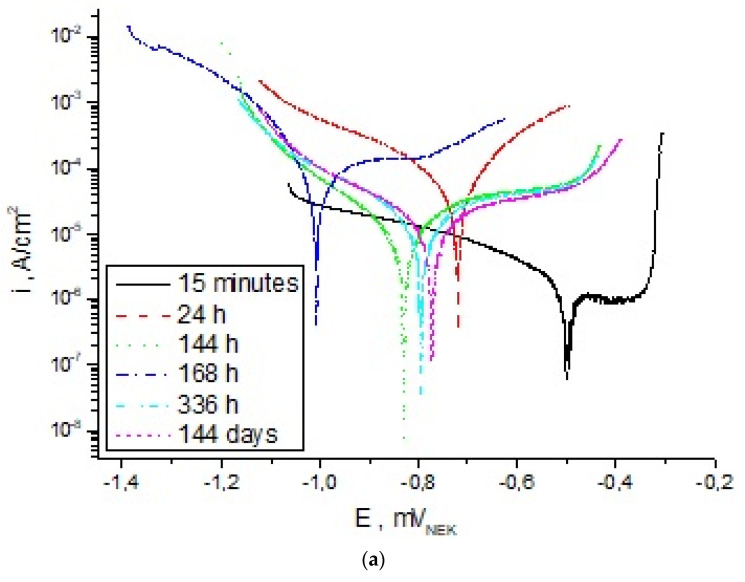

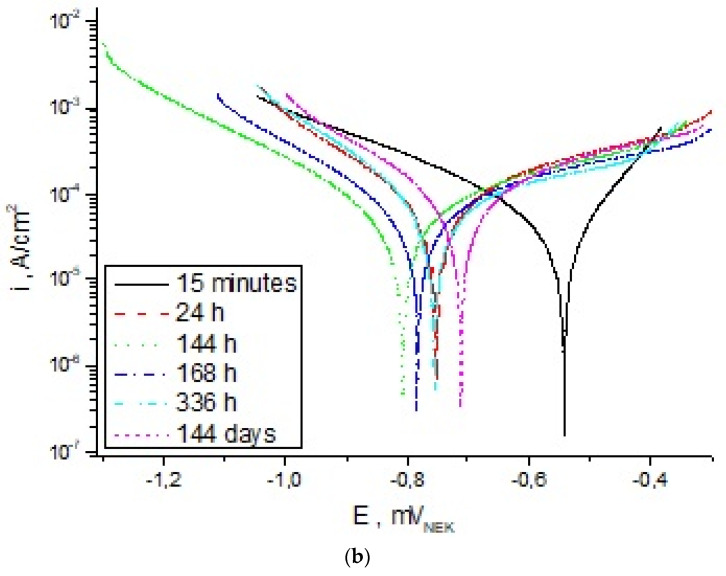
Polarization curves after different holding times of samples in a 3% aqueous NaCl solution: (**a**) cast steel L120G13, (**b**) composite L120G13 + Al_2_O_3_.

The course of the polarisation curves of cast steel L120G13 (Figure 6a) and the composite L120G13 (Figure 6b) indicates a shift of the cathode-anode transition potential (E_K-A_) towards more negative values as a result of the action of 3% NaCl. Thus, the proportion of electrode processes on these materials is different. In the case of cast steel, the greatest changes in the E_K-A_ value (approx. 300 mV) were found after 1 day of exposure in a corrosive solution (Table 4, Figure 6a). For L120G13 with Al_2_O_3_, the difference in cathode-anode transition potential value after this exposure time was approx. 210 mV (Table 4). Extending the exposure time did not cause such significant changes in E_K-A_ values for both cast steel and composite (Table 4, Figure 6).

The course of cathode curves for cast steel L120G13 shows differences in their shape and current densities, which may result from the intensity of electrode processes taking place on the surface of the samples (Figure 6a). The shape of the individual curves, both cathode and anode, as well as the current density in the anode area after different exposure times of the composite L120G13 with Al_2_O_3_ in the corrosive solution are comparable (Figure 6b). The presence of corrosion products on the surface of cast steel and composite reduces the speed of electrode processes—this is indicated by the gentle course of these curves after long-term exposure compared to rapid changes in the current density in the anode area for cast steel after 15-min exposure to 3% NaCl (Figure 6).

Changes in the course of the potentiodynamic curves are reflected in the values of the polarisation resistance (R_p_) and corrosion current density (i_corr_)—Table 4. The R_p_ value of cast steel after a 15-min exposure to 3% NaCl was approx. 1880 kΩ· cm^2^, and the rate of corrosion was approx. 1.4 × 10^−6^ A/cm^2^. Introduction of Al_2_O_3_ to the cast steel matrix causes a 43% reduction in polarisation resistance and an increase in the corrosion rate to the value of 2.4 × 10^−5^ A/cm^2^ (Table 4). When exposed to a corrosive solution, both cast steel and composite retain high stability. After 144-day exposure in a 3% NaCl solution, the differences in the polarisation resistance of cast steel and composite are small—approx. ok. 75 kΩ × cm^2^. The corrosion rate of the composite is less than an order of magnitude higher compared to cast steel—Table 4.

Based on the determined values of the polarisation resistance and the corrosion rate, it can be concluded that the corrosion resistance of the composite L120G13 + Al_2_O_3_ is not significantly lower than that of the cast steel itself. It is very important from the application point of view, because the introduction of Al_2_O_3_ to cast steel allows for a radical improvement in mechanical properties without deteriorating the corrosion resistance.

### 3.3. Microscopic Observations of Samples after Corrosion Tests

#### 3.3.1. Observations of the Surface of Samples

**Figure 7:**
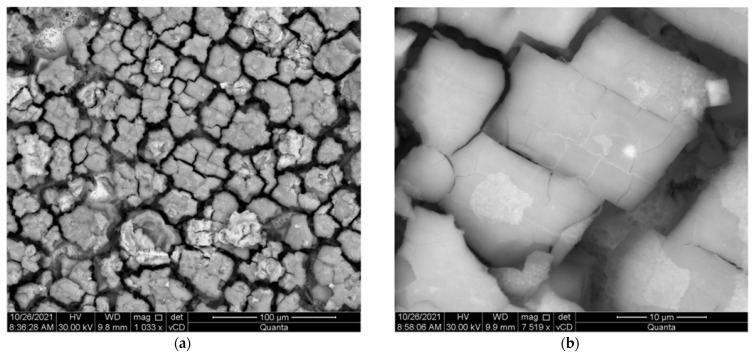
The surface of: (**a**) L120G13 cast steel after 144 days of being kept in a 3% aqueous NaCl solution with a layer of corrosion products, (**b**) an enlarged fragment of the area shown in (**a**).

**Figure 8:**
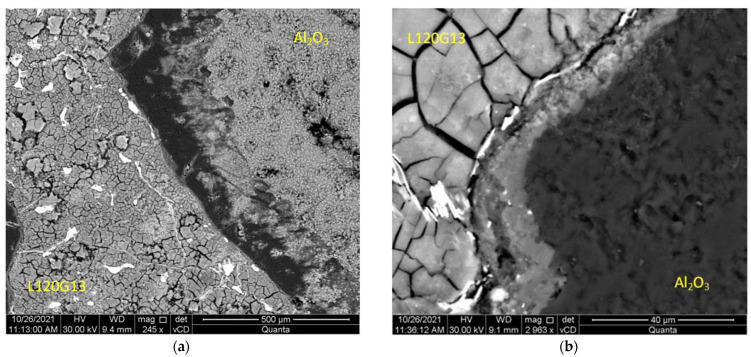
The surface of: (**a**) L120G13 + Al_2_O_3_ composite after 144 days of storage in a 3% aqueous NaCl solution with a layer of corrosion products, (**b**) an enlarged fragment of the area shown in (**a**).

In the next stage of research, a series of microscopic observations by SEM (BSE imaging technique was used) of the surface of the samples after potentiodynamic tests and their previous 144-day storage in a corrosive solution were carried out. On the surface of cast steel L120G13 and composite L120G13 + Al_2_O_3_, the formation of a layer of corrosive products with a fairly even distribution was observed—Figure 7 and Figure 8.

**Figure 9:**
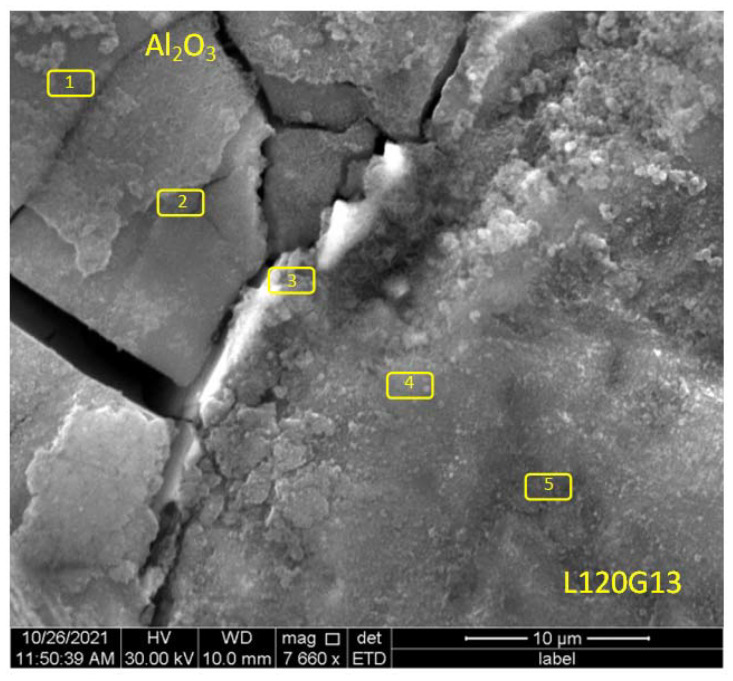
The surface of the L120G13 + Al_2_O_3_ composite with a layer of corrosion products after 144 days in a 3% corrosive solution with EDS measurement points marked.

The EDS analysis carried out on the surface of the composite samples shows the ability to passivate them due to the formation of oxides on their surface, including Cr, Mn, Ni (Figure 9, Table 5). Figure 9 presents a photo showing the area of contact of both components of the composite with the marked areas of EDS measurements. A WDS detector was used for comparison purposes. The results of the measurements are presented in Table 5.

After thorough cleaning of corrosion products, the surface topography of the samples was measured. The results are shown in Table 6.

**Figure 10:**
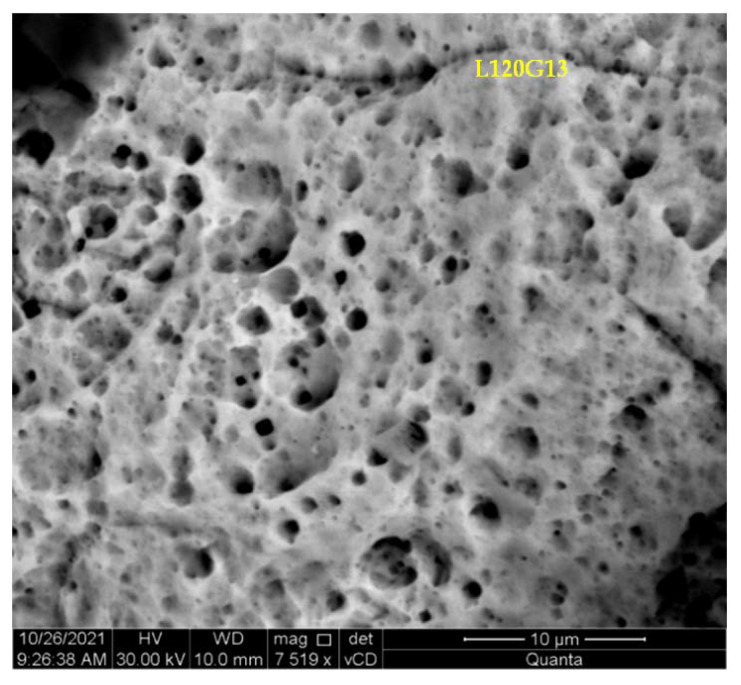
The surface of the composite sample showing a matrix fragment after cleaning it from corrosion products.

Figure 10 shows a fragment of the composite surface observed in SEM with clearly visible corrosion damage. Microscopic observations revealed that the damage was of uniform corrosion character with relatively shallow pits. This confirms the increased corrosion resistance of the tested material.

**Figure 11:**
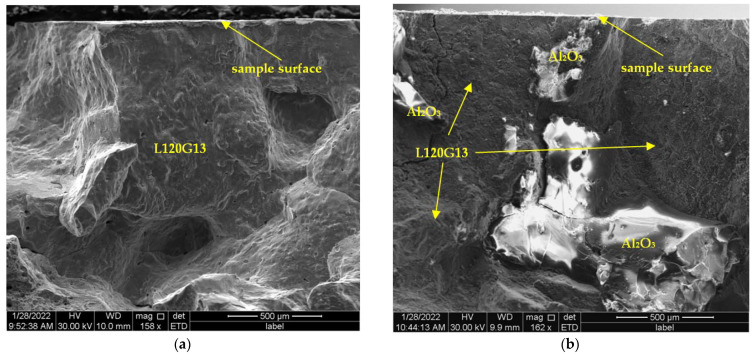
Sample fracture area near the edge after freezing and fracture. Visible features of the plastic fracture: (**a**) cast steel L120G13, (**b**) composite L120G13 + Al_2_O_3_.

After corrosion tests, samples of cast steel L120G13 and composite L120G13 + Al_2_O_3_ were frozen in liquid nitrogen and then broken. The images of the fracture surfaces subjected to intense and long-lasting action of a corrosive solution are presented in Figure 11. No signs of intercrystalline corrosion were found. There were also no signs of corrosion damage at the boundary of the cast steel matrix and corundum particles. It can therefore be concluded that the corrosion processes only took place on the surface of the samples and that they were characterised by a relatively even distribution. This is very advantageous from the point of view of corrosion resistance, especially from the point of view of the service life of the composite.

#### 3.3.2. Observations of Breakthroughs Samples

**Figure 12:**
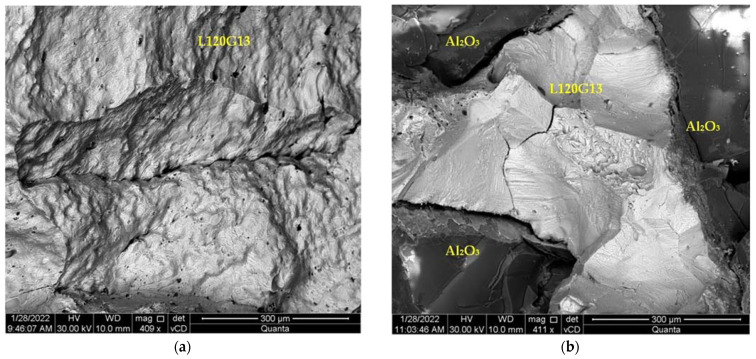
The fracture surface of the samples near the core after freezing and fracture. Visible features of the plastic fracture: (**a**) cast steel L120G13, (**b**) composite L120G13 + Al_2_O_3_.

The samples were broken immediately after freezing in liquid nitrogen, then their breakthroughs were monitored by SEM. The fractures shown in Figure 11 and Figure 12 do not show the features typical of brittle fracture. After freezing, the samples were still highly resistant to cracking. This issue will be the subject of further detailed studies.

## 4. Conclusions

On the basis of the obtained results, it can be concluded that the strengthening of the L12G13 cast steel with Al_2_O_3_ alumina macromolecules in order to significantly improve the abrasive wear resistance does not significantly deteriorate the corrosion resistance.

SEM tests have shown that at the interface between cast steel and corundum, a permanent diffusion layer is formed, in the area of which manganese shows simple segregation, while with iron and chromium segregation is reversed. At the same time, these elements, passing from the liquid alloy to the top layer of corundum particles, push aluminium and oxygen inside the corundum grains.

Corrosion tests indicate comparable corrosion resistance of cast steel L120G13 and composite L120G13 + Al_2_O_3_. No intercrystalline corrosion was found on the surface of the composite matrix. On the surface of the composite, no signs of pitting corrosion were found in the areas of contact between the matrix and the corundum (a phenomenon important from the point of view of its operational durability).

The breaking tests after potentiodynamic corrosion measurements and freezing in liquid nitrogen showed high durability of both cast steel L120G13 and the composite L120G13 + Al_2_O_3_.

## Figures and Tables

**Table 1:** Results of the analysis of the chemical composition of EDS in the areas where corundum and cast steel meet.

Measurement No.	Chemical Composition (%_mas_)														Ratio Al/O
	L120G13								Al_2_O_3_						
	Fe	C	Si	Mn	Cr	Ni	P	S	Al	O	Ti	Si	Mg	Ca	-
1	81.72	1.35	0.77	13.95	1.24	0.85	0.04	0.08	-	-	-	-	-	-	-
2	83.38	1.45	0.67	12.54	1.03	0.81	0.05	0.07	-	-	-	-	-	-	-
3	86.77	1.18	0.73	9.03	1.15	1.01	0.04	0.09	-	-	-	-	-	-	-
4	12.58	-	-	10.04	0.96	-	-	-	37.96	37.99	-	0.24	0.16	0.07	0.99
5	3.45	-	-	12.07	0.69	-	-	-	43.32	39.71	0.73	-	-	0.03	1.09
6	0.55	-	-	15.01	0.24	-	-	-	43.38	40.69	0.07	-	0.06	-	1.06
7	-	-	-	3.05	-	-	-	-	51.03	45.77	-	0.08	-	0.07	1.11
8	-	-	-	-	-	-	-	-	53.08	46.55	0.24	0.13	-	-	1.14

**Table 2:** Results of EDS analysis of chemical composition in the contact area between corundum grain and cast steel.

Measurement No.	Chemical Composition (%_mas_)						Ratio Al/O
	Al	O	Ti	Si	Mg	Ca	
1	53.86	45.23	0.72	-	0.12	0.07	1.19
2	52.96	47.00	0.31	0.15	-	-	1.13
3	51.81	46.03	0.92	-	0.09	-	1.13
4	54.21	45.31	-	0.23	0.14	0.11	1.19
5	53.45	45.72	0.71	-	-	0.12	1.17

**Table 3:** Corrosion rate V_P_ after exposure of specimens raw and soaked in 3—% solution of NaCl.

Cast	V_P_ (mm/Year) after Exposure for Specified Time (Days)						
	1	2	5	8	13	18	25
L120G13	0.56	0.59	0.55	0.52	0.45	0.44	0.38
L120G13 + Al_2_O_3_	0.59	0.60	0.54	0.50	0.48	0.40	0.36

**Table 4:** Electrochemical indices characterising corrosion processes.

Cast	Time of Exposure to 3% NaCl	E_OC_ (mV)	E_K-A_ (mV)	i_corr_ (μA/cm^2^)	R_p_ (kΩ × cm^2^)
L120G13	15 min	−566.4	−496.0	1.39 ×10^−6^	1878.1
	1 day	−667.0	−793.0	9.57 ×10^−6^	2724.0
	6 days	−668.0	−832.0	7.64 × 10^−6^	3411.0
	7 days	−641.0	−861.0	6.31 × 10^−6^	413.6
	14 days	−675.0	−770.0	5.96 × 10^−6^	2669.0
	144 days	−676.0	−719.0	4.07 × 10^−6^	641.2
L120G13 + Al_2_O_3_	15 min	−546.0	−542.0	2.41 × 10^−5^	1083.0
	1 day	−545.0	−753.0	3.44 × 10^−5^	756.0
	6 day	−552.0	−808.0	2.43 × 10^−5^	1069.0
	7 days	−564.0	−781.0	2.64 × 10^−5^	987.0
	14 days	−551.0	−710.0	4.01 × 10^−5^	651.9
	144 days	−588.0	−751.0	3.63 × 10^−5^	717.5

**Table 5:** The results of the analysis of the chemical composition of EDS in the areas of contact between cast steel and corundum.

Measurement No.	Chemical Composition (%_mas_)													
	L120G13 + Al_2_O_3_													
	Fe	C	Si	Mn	Cr	Ni	P	S	Al	O	Ti	Si	Mg	Ca
1	45.97	1.33	-	3.14	6.50	1.23	0.08	-	-	40.78	0.07	0.80	-	0.10
2	46.41	1.48	0.63	2.98	5.45	1.15	0.05	-	-	41.05	0.08	0.63	-	0.09
3	30.88	1.41	0.99	3.55	2.16	0.22	0.07	-	6.84	52.47	0.86	0.56	-	1.25
4	1.22	-	-	1.59	-	-	-	-	51.23	45.96	0.97	-	-	0.07
5	1.40	-	-	-	-	-	-	-	52.81	44.84	0.87	0.02	0.01	0.06

**Table 6:** Indicators determining the surface topography of samples after potentiodynamic tests.

Cast	Casting Surface Topography Indicator (μm)		
	R_pAVR_	R_vAVR_	R_zAVR_
L120G13	4.15	6.82	10.97
L120G13 + Al_z_O_3_	5.13	6.50	11.63

## Data Availability

Not applicable.
